# Prevalence and factors associated with the co-occurrence of health risk behaviors in adolescents

**DOI:** 10.1016/j.rpped.2015.02.002

**Published:** 2015

**Authors:** Anísio Luiz da Silva Brito, Carla Meneses Hardman, Mauro Virgílio Gomes de Barros

**Affiliations:** a Universidade de Pernambuco (UPE), Recife, PE, Brazil; b Universidade Federal de Santa Catarina (UFSC), Florianópolis, SC, Brazil

**Keywords:** Risk behaviors, Adolescent, Epidemiology

## Abstract

**Objective:**

To analyze the prevalence and factors associated with the co-occurrence of health risk behaviors in adolescents.

**Methods:**

A cross-sectional study was performed with a sample of high school students from state public schools in Pernambuco, Brazil (*n*=4207, 14-19 years old). Data were obtained using a questionnaire. The co-occurrence of health risk behaviors was established based on the sum of five behavioral risk factors (low physical activity, sedentary behavior, low consumption of fruits/vegetables, alcohol consumption and tobacco use). The independent variables were gender, age group, time of day attending school, school size, maternal education, occupational status, skin color, geographic region and place of residence. Data were analyzed by ordinal logistic regression with proportional odds model.

**Results:**

Approximately 10% of adolescents were not exposed to health risk behaviors, while 58.5% reported being exposed to at least two health risk behaviors simultaneously. There was a higher likelihood of co-occurrence of health risk behaviors among adolescents in the older age group, with intermediate maternal education (9-11 years of schooling), and who reported living in the driest (semi-arid) region of the state of Pernambuco. Adolescents who reported having a job and living in rural areas had a lower likelihood of co-occurrence of risk behaviors.

**Conclusions:**

The findings suggest a high prevalence of co-occurrence of health risk behaviors in this group of adolescents, with a higher chance in five subgroups (older age, intermediate maternal education, the ones that reported not working, those living in urban areas and in the driest region of the state).

## Introduction

Over the past decades, exposure to health risk behaviors has become one of the most widely investigated subjects in studies with young populations.^1^^,^ 2 The interest in investigations focusing on this subject can be explained, at least in part, by the fact that such behaviors can be established and incorporated into the lifestyle at an early age,^3^^,^ 4 and due to their connection with biological risk factors^5^ and the presence of established metabolic or cardiovascular disease (CVD).^6^

The prevalence of co-occurrence of health risk behaviors in adolescents has been described in several studies.^7^^-^ 17 However, it was observed that the studies developed in Brazil, except for the survey performed by Farias Júnior et al.,^15^ relied on very specific samples: laboratory school students^17^ and day-shift students from public schools in a city from southern Brazil.^16^ Therefore, the results of these studies cannot be extrapolated to other regions of the country due to socioeconomic and cultural contrasts, which are known to differentiate the exposure to health risk behaviors, as reported by Nahas et al.^18^

Epidemiological surveys on the co-occurrence of health risk behaviors in adolescents and their associated factors can help to identify risk groups and to monitor the health levels of this population, which can support the development of public policies to promote health, using earlier intervention strategies to prevent these habits. Thus, the aim of this study was to analyze the prevalence and factors associated with co-occurrence of health risk behaviors in adolescents.

## Method

This is a secondary analysis of data from a cross-sectional epidemiological survey, school-based and statewide (state of Pernambuco, Brazil), called "Lifestyle and health risk behaviors in adolescents: from prevalence study to intervention". The research protocol was approved by the Institutional Review Board of Hospital Agamenon Magalhães, in compliance with the standards established in Resolutions 196 and 251 by the National Health Council.

The target population, estimated at 352,829 individuals, according to data from the Education and Culture Secretariat of the State of Pernambuco, consisted of high-school students enrolled in public schools, aged 14-19 years. The following parameters were used to calculate sample size: 95% confidence interval; sampling error of 3% points; prevalence estimated at 50% (this option was chosen based on the multiple factors analyzed in the study), and the effect of sample design, established at four times the minimum sample size. Based on these parameters, the calculated sample size was 4217 students.

Considering the sampling process, we tried to ensure that the selected students represented the target population regarding the geographic regions (metropolitan area, forest area [*Zona da Mata*], arid [*Agreste*], semi-arid [*Sertão*] and semi-arid region of the Sao Francisco river [*Sertão do São Francisco*]), school size and shift (daytime/nighttime). The regional distribution was analyzed based on the number of students enrolled in each of the 17 GEREs (*Gerências Regionais de Ensino* - Regional Education Administration). Schools were classified according to the number of students enrolled in high school, according to the following criteria: small - less than 200 students; medium - 200-499 students, and large - 500 students or more. Students enrolled in the morning and afternoon periods were grouped into a single category (daytime students). All students in the selected classes were invited to participate.

We used cluster sampling in two stages, using the school and class as the primary and secondary sampling units, respectively. In the first stage, we performed the random selection of the schools, aiming to include at least one school of each size by GERE. In the second stage, 203 classes were randomly selected among those existing in the schools selected in the first stage.

Data collection was performed using an adapted version of the Global School-Based Student Health Survey (GSHS) questionnaire. This tool had both face and content validity evaluated by experts (researchers experienced in performing epidemiological studies focused on health behaviors), and had its indicators of co-occurrence validity and reproducibility tested in a pilot study. Consistency indicators of test-retest measure ranged from moderate to high (kappa coefficient =0.52-1.00)^19^^-^ 21 for most items. The test-retest reproducibility coefficients (kappa coefficient) of the measures used in this study were: 0.86 for physical activity; 0.66 for the consumption of fruits; 0.77 for the consumption of vegetables; 0.76 for alcohol consumption; 0.62 for tobacco use, and 0.74 for sedentary behavior.

Data collection was carried out from April to October 2006. The questionnaires were applied in the classroom. The students were advised by two previously trained applicators, which clarified and assisted in filling out the data. All students were informed that their participation was voluntary and that the questionnaires did not contain any personal identification. Students were also informed that they could leave the study at any stage of data collection. A passive informed consent form was used to obtain the permission of parents for students younger than 18 years to participate in the study. Participants aged 18 or older signed the term, indicating their agreement to participate in the study.

The dependent variable (co-occurrence of health risk behaviors) was obtained from the sum of five risk behaviors: low level of physical activity (<300 min/week); sedentary behavior (>4 h/day); occasional consumption of fruits and vegetables (<once a day); alcohol consumption (having consumed alcohol in the last 30 days), and smoking (having smoked in the last 30 days). These factors were chose because they are lifestyle modifiable factors that appear to be more strongly associated with non-communicable chronic diseases, and represent the highest global burden of disease and mortality worldwide.^22^ Sedentary behavior was included because it is treated as a distinct behavior from low levels of physical activity, and it has a high prevalence in the population, in addition to being an important impact on adolescent health.^23^ Information regarding the description of these variables can be found in previous studies.^19^^-^ 21 The obtained responses resulted in an outcome with zero (no risk factor present) to five identified risk behaviors. Subsequently, for analysis purposes, the occurrence of risk behaviors was divided in four categories (0, 1, 2, ≥3). The independent variables were: gender; age (14-16 or 17-19 years); school shift (daytime or nighttime); school size (small, medium or large); maternal education (low: ≤8; intermediate: 9-11, or high: ≥12 years); occupational status (working/not working); ethnicity (Caucasian/non-Caucasian); geographic region (metropolitan, forest area or semi-arid) and place of residence (urban and rural).

The data tabulation procedure was carried out in a database created with the EpiData Entry software (version 3.1). To perform the analysis, Stata software (version 10) was used. In the bivariate analysis, the chi-square test was used for heterogeneity and for trend to determine the prevalence of co-occurrence of health risk behaviors by categories of the independent variables.

To evaluate possible associations between independent and dependent variables, an analysis of ordinal logistic regression was performed with a proportional odds model. The assumption of proportionality was assessed by the likelihood ratio test, and the significance of coefficients, by the Wald test. Analyses were carried out in two stages: first, by making simple regressions of the independent variables in relation to the outcome. Then, a multivariate analysis was performed to determine whether the demographic and school-related factors were associated or not with the outcome. All independent variables entered the multivariate model at the same level of analysis and were excluded by stepwise method with backward elimination, using a *p*-value <0.2 as an exclusion criterion of variables during the modeling stages. These results are shown as odds ratios and respective confidence intervals.

After selecting the variables that would comprise the regression model, we tested the existence of possible collinearity between the geographic region (metropolitan, forest area or semi-arid region) and place of residence (urban and rural) covariates, and no linear association (variance inflation factor (VIF) values <10) was identified between these two variables.

## Results

Of the total of adolescents attending the selected classes in 76 assessed schools (4269), 55 refused to participate in the study, and seven were excluded due to incomplete or inconsistent data in the questionnaire. The final sample consisted of 4207 adolescents (59.8% girls), aged between 14 and 19 years (mean 16.8 years, SD=1.4). Other sample characteristics are shown in Table 1. Among the analyzed variables in the study, with the exception of maternal education (6.1%), the rate of unanswered questions did not exceed 2.0%.

**Table 1:** Sample characteristics by gender.

Variable	All			Boys			Girls	
	%	*n*		%	*n*		%	*n*
*Age range*									
14–16 years	42.0	1,766		35.4	598		46.4	1,165
17–19 years	58.0	2,441		64.6	1,089		53.6	1,346

*School shift*									
Daytime (morning/afternoon)	57.6	2,414		53.9	908		60.0	1,506
Nighttime	42.4	1,780		46.1	778		40.0	1,002

*Maternal schooling*									
≤8	72.5	2,865		69.4	1,086		74.5	1,771
9–11	21.1	833		22.5	352		20.2	480
≥12	6.4	253		8.1	127		5.30	126

*Ethnicity*									
Caucasian	25.2	1,057		24.8	417		25.5	639
Non-Caucasian	74.8	3,136		75.2	1,262		74.5	1,866

*Geographic region*									
Metropolitan	41.8	1,757		39.8	670		43.2	1,084
Forest area	17.7	743		18.1	306		17.3	434
Semi-arid	40.6	1,707		42.1	711		39.5	993

*Residence area*									
Urban	79.0	3,294		78.1	1,311		79.5	1,983
Rural	21.0	877		21.9	367		20.5	510

*School size*									
Small	8.9	376		9.0	152		8.80	221
Medium	25.8	1,084		27.0	456		25.0	628
Large	65.3	2,747		64.0	1,079		66.2	1,662

*Employment status*									
Unemployed	78.5	3,276		69.2	1,157		84.8	2,119
Employed	21.5	895		30.8	514		15.2	381
Low level of physical activity	65.1	2,731		57.5	971		70.2	1,754
Exposure to sedentary behavior	18.7	782		16.5	277		20.2	504
Occasional consumption of fruit and/or vegetables	51.4	2,145		53.5	893		50.0	1,248
Alcohol consumption	30.4	1,273		38.6	648		24.8	622
Smoking	7.6	320		9.8	165		6.2	155

**Figure 1:**
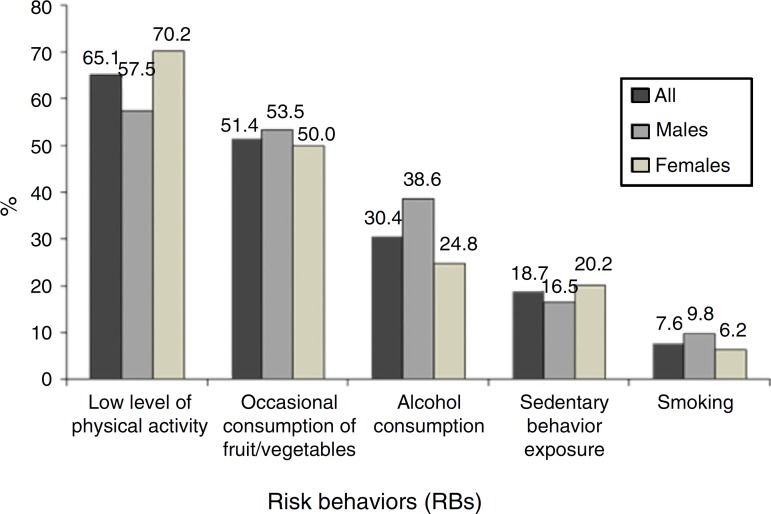
Prevalence of health risk behaviors in high-school adolescents in the state of Pernambuco, Brazil, 2006.

**Figure 2:**
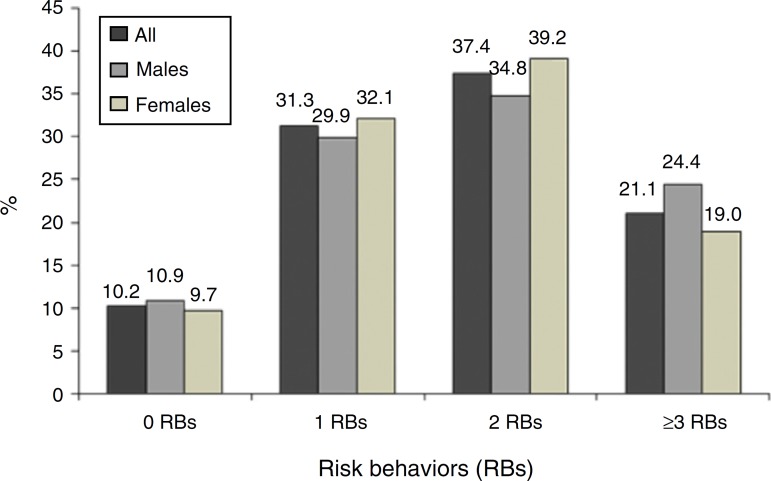
Prevalence of co-occurrence of health risk behaviors among high-school adolescents in the state of Pernambuco, Brazil, 2006.

Fig. 1 shows the prevalence of exposure to the five health risk behaviors targeted in this study. The results for these behaviors will not be explored in this study, as they already have been presented alone in previous investigations.^19^^-^ 21 Fig. 2 shows the prevalence of co-occurrence of health risk behavior exposure observed in the sample by gender.

In the bivariate analysis, we observed that the proportion of adolescents simultaneously exposed to three or more risk behaviors was statistically higher among older students (17-19 years), adolescents with higher maternal education, students living in the urban area and those who lived in the semi-arid region when compared to their peers (Table 2).

**Table 2:** Prevalence of co-occurrence of health risk behaviors according to demographic, socioeconomic, school-related and regional division variables in high-school adolescents from Pernambuco, 2006.

Variables	Co-occurrence of health risk behaviors (RBs)											
	0 RBs			1 RBs			2 RBs			≥3 RBs		*p*-value
	%	*n*		%	*n*		%	*n*		%	*n*	
**Demographic factors**												
*Gender*												
Male	10.9	180		29.9	491		34.8	571		24.4	400	<0.083
Female	9.7	240		32.1	794		39.2	968		19.0	470	
*Age*												
14–16 years	10.5	183		32.2	560		38.3	666		19.0	331	0.028
17–19 years	9.9	237		30.6	730		36.8	876		22.7	540	
*Maternal schooling*												
≤8 years	9.9	277		32.3	907		38.8	1,091		19.0	535	0.008
9–11 years	11.2	92		28.6	234		33.5	274		26.7	219	
≥12 years	11.2	28		26.5	66		35.7	89		26.5	66	
*Ethnicity*												
Caucasian	8.8	91		33.8	350		35.4	366		22.0	227	0.732
Non-Caucasian	10.6	326		30.4	934		38.1	1172		20.9	644	
*Geographic region*												
Metropolitan	12.0	207		32.0	551		35.3	609		20.7	356	<0.001
Forest area	9.5	69		33.7	246		40.6	296		16.2	118	
Semi-arid	8.6	144		29.5	493		38.1	637		23.8	397	
*Residence area*												
Urban	10.1	328		30.8	994		36.6	1181		22.5	728	0.009
Rural	9.9	86		33.7	292		40.4	350		15.9	138	

**School-related factors**												
*School shift*												
Daytime	10.6	252		31.5	749		36.6	870		21.3	507	0.522
Nighttime	9.7	168		31.1	541		38.4	669		20.8	363	
*School size*												
Small	10.6	39		27.7	102		39.4	145		22.3	82	0.085
Medium	11.0	117		35.6	378		35.2	374		18.2	193	
Large	9.8	264		30.1	810		38.0	1023		22.1	596	

**Socioeconomic factors**												
*Employment status*												
Unemployed	9.6	308		31.5	1017		37.8	1219		21.1	682	0.209
Employed	12.5	109		30.2	263		36.0	313		21.3	186	

Table 3 shows the results of the ordinal logistic regression analysis for the co-occurrence of health risk behaviors according to demographic and school-related factors. In the adjusted analysis, it was observed that age, occupational status, maternal education, geographic region and place of residence were statistically associated with higher co-occurrence of health risk behaviors.

**Table 3:** Ordinal logistic regression for co-occurrence of health risk behaviors and demographic, socioeconomic, school-related and regional division variables in high-school adolescents from Pernambuco, 2006.

Variables	Co-occurrence of health risk behaviors					
	Crude OR	95%CI	*p*	Adjusted OR	95%CI	*p*
*Gender*						
Male	1			1		
Female	0.90	0.80–1.00	0.060	0.91	0.81–1.03	0.140

*Age*						
14–16 years	1			1		
17–19 years	1.13	1.01–1.27	0.028	1.17	1.04–1.32	0.008

*Maternal schooling*						
≤8 years	1		0.004	1		0.009
9–11 years	1.21	1.05–1.40	0.010	1.21	1.04–1.40	0.011
≥12 years	1.26	0.99–1.60	0.058	1.21	0.95–1.55	0.121

*Ethnicity*						
Caucasian	1			Excluded		
Non-Caucasian	0.99	0.87–1.13	0.927			

*Geographic region*						
Metropolitan	1			1		
Forest area	0.97	0.83–1.14	0.731	1.07	0.90–1.26	0.435
Semi-arid	1.27	1.13–1.44	<0.001	1.39	1.22–1.59	<0.001

*Residence area*						
Urban	1			1		
Rural	0.83	0.73–0.95	0.008	0.78	0.68–0.91	0.001

*School shift*						
Daytime	1			Excluded		
Nighttime	1.04	0.93–1.16	0.542			

*School size*						
Small	1		0.084	Excluded		
Medium	0.76	0.61–0.94	0.013			
Large	0.97	0.79–1.19	0.782			

*Employment status*						
Unemployed	1			1		
Employed	0.93	0.81–1.06	0.285	0.86	0.74–0.99	0.040

It was verified that older adolescents (17-19 years) had a 17% higher chance of simultaneous exposure to more than three health risk behaviors when compared to younger ones. Students who reported working had a 14% lower chance of having more than three risk behaviors when compared to those who did not work. On the other hand, adolescents who reported mothers with intermediate education (9-11 years) had a 21% higher chance of having co-occurrence of risk behaviors, compared to those who reported lower maternal education (≤8 years).

The chance of co-occurrence of a higher number of health risk behaviors was 22% lower among adolescents who reported residing in rural areas, when compared to those living in urban areas. Adolescents who reported living in the semi-arid region showed a 39% higher chance of exposure to multiple health risk behaviors when compared to adolescents living in the metropolitan area.

## Discussion

The results of this study show that the prevalence of simultaneous exposure to health risk behaviors among adolescents from the state of Pernambuco was high, as observed in similar studies.^8^^,^ 10 ^-^ 12 Another important result was the identification of five significant factors associated to the higher co-occurrence of these behaviors, namely: age range, maternal education, geographic region, working status, and place of residence.

The results of this survey indicated that 58.5% of adolescents were simultaneously exposed to two or more risk behaviors, as observed in a study carried out in the city of João Pessoa, state of Paraiba.^15^ The importance of this finding lies in the fact that health problems can be caused by a set of aggregated risks behaviors, such as throat cancer, which can be explained by the simultaneous occurrence of two habits (smoking and alcohol consumption), as highlighted by the World Health Organization (WHO).^24^

In this study, simultaneous exposure to a higher number of health risk behaviors was higher among older adolescents. As seen in the available studies, the prevalence of simultaneous exposure to health risk behaviors increases with age.^8^^,^ 13 ^,^ 25 That can be explained by the fact that adolescents acquire greater autonomy and social and economic independence with age,^26^ favoring access to places that sell alcoholic beverages, cigarettes and other drugs.

It is worth mentioning in this study the association between intermediate maternal education (9-11 years) and higher co-occurrence of health risk behaviors among adolescents. This is an interesting fact, because the higher the educational level of the mother, supposedly the better understanding she would have on the benefits of having a healthier life style, and therefore would have a greater chance of providing more support to her children.^27^ One of the possible explanations lies in the fact that higher levels of education are seen among those mothers who probably work out of their households and, therefore, spend less time with their adolescent children.

It was also observed that adolescents who reported having a job had lower chances of simultaneous exposure to a higher number of health risk behaviors, when compared to those who did not work. In a society where young individuals face great challenges to enter the labor market, it is possible to assume that young individuals who engage in some labor activity have higher self-esteem, autonomy and personal responsibility, characteristics that may favor the adoption of healthier behaviors.

Adolescents who live in the semi-arid region of Pernambuco showed a 39% increase in the chance of simultaneous exposure to a higher number of health risk behaviors compared to their peers living in the metropolitan area. Comparative studies with analysis of simultaneous exposure to lifestyle habits are scarce, making the comparisons impossible. However, Matsudo et al.^28^ carried out a study in the state of São Paulo, observing that the individuals who lived on the coast were more active than those living in the countryside. This may be related to the low supply of leisure and physical facilities for physical activities in the countryside. Moreover, it may be related to the availability, accessibility and quality of food preservation in this region, where there is an acknowledged shortage of water resources, indispensable for both the production and the processing of fresh food.

On the other hand, adolescents who live in rural areas had a 22% decrease in the chance of simultaneous exposure to a higher number of health risk behaviors when compared to those living in urban areas. This can be explained by the specific characteristics of the types of activities carried out in rural areas, which require greater energy expenditure to be performed (e.g. extensive and family farming, livestock, vegetal extractivism, mineral extractivism, etc.),^29^ in addition to greater access to foods such as cereals and derivatives (beans, rice and corn) and tubers (potatoes, cassava and others), which are essentially products of family agriculture, as well as the lower access to ready-made meals and industrialized mixes.^30^

The lack of similar studies makes it difficult to compare the findings of the present study. What was found in the literature was limited to studies that evaluated the association of these factors with isolated exposure to one or another risky behavior. Similar studies available^13^^-^ 17 used very different methodological procedures, particularly regarding the type, quantity and definition of characterizing risk variables.

The generalization of the results of this study must be made with caution, as only adolescents attending public schools were included. One can assume that the results are different in samples of adolescents attending private schools and among those who are not enrolled in the formal educational system. On the other hand, the decision to not include private schools in the sampling planning was due to the fact that more than 80% of adolescents from Pernambuco were enrolled in public schools.

It is noteworthy that the prevalence shown in this article discloses a scenario observed some time ago and, therefore, the interpretation of these parameters should be made carefully, as social and demographic changes that have occurred in the Brazilian northeast region during this period may have affected these indicators. On the other hand, it is not plausible to assume that the associations that were identified and reported in this study would be different due to possible changes in the prevalence of some factor.

Despite the good reproducibility levels of the tool, one cannot rule out the possibility of information bias, as adolescents tend to overestimate or, at other times, underestimate the exposure to risk behaviors.

However, the findings of this survey add important evidence to the available body of knowledge on the prevalence and factors associated with co-occurrence of health risk behaviors in adolescents. Additionally, the study was performed with a relatively large sample, representative of high-school students from public schools in the state of Pernambuco. It is believed that the evidence shown in this study may help identify more vulnerable subgroups, thus contributing to decision-making and appropriate intervention strategy planning. Moreover, it can lead to the development of other investigations.

Considering these findings, it can be concluded that there is a large portion of adolescents exposed to simultaneous health risk behaviors. It was also verified that older adolescents, with mothers of intermediate educational levels and living in the semi-arid region had higher chance of simultaneous exposure to a higher number of health risk behaviors, thus configuring higher-risk subgroups, whereas adolescents who worked and those living in rural areas were less likely to have simultaneous exposure to a higher number of health risk behaviors.
