# Supplementary material for: Prevalence and factors associated with the co-occurrence of health risk behaviors in adolescents
Source: Rev Paul Pediatr. 2015 Oct-Dec;33(4):423–30. doi: 10.1016/j.rpped.2015.02.002 (PMC4685562; doi:10.1016/j.rpped.2015.02.002)
Supplement: Translation

# Translation: Prevalência e fatores associados à simultaneidade de comportamentos de risco à saúde em adolescentes

**Authors:** Anísio Luiz da Silva Brito, Carla Meneses Hardman, Mauro Virgílio Gomes de Barros

**Affiliations:** a Universidade de Pernambuco (UPE), Recife, PE, Brasil; b Universidade Federal de Santa Catarina (UFSC), Florianópolis, SC, Brasil

**Keywords:** Comportamentos de risco, Adolescente, Epidemiologia

## Abstract

**Objetivo:**

Analisar a prevalência e os fatores associados à simultaneidade de comportamentos de risco à saúde em adolescentes.

**Métodos:**

Estudo transversal feito com amostra representativa de estudantes do ensino médio da rede pública estadual de Pernambuco (n=4.207, 14-19 anos). Os dados foram obtidos mediante questionário. A simultaneidade de comportamentos de risco à saúde foi determinada pelo somatório da exposição dos adolescentes a cinco comportamentos de risco (baixo nível de atividade física, comportamento sedentário, consumo ocasional de frutas/hortaliças, consumo de bebidas alcoólicas e tabagismo). As variáveis independentes foram sexo, faixa etária, turno, porte da escola, escolaridade materna, situação ocupacional, cor da pele, região geográfica e local de residência. Os dados foram analisados por meio de regressão logística ordinal com modelo de *odds* proporcionais.

**Resultados:**

Aproximadamente 10% dos adolescentes não estavam expostos a comportamentos de risco, enquanto 58,5% apresentaram dois ou mais comportamentos de forma simultânea. Verificou-se elevação da simultaneidade de comportamentos de risco com aumento da faixa etária, com escolaridade materna intermediária (9-11 anos de estudo) e residência na Região Semiárido. Adolescentes que referiram trabalhar e aqueles que residiam em área rural apresentaram menor chance de simultaneidade de comportamentos de risco.

**Conclusões:**

Os achados indicam uma alta prevalência de exposição simultânea a comportamentos de risco à saúde nesse grupo de adolescentes. Identificaram-se cinco subgrupos de maior chance de exposição (faixa etária mais alta, escolaridade materna intermediária, aqueles que referiram não trabalhar, residir em área urbana e na Região Semiárido).

## Introdução

Ao longo das últimas décadas, a exposição a comportamentos de risco à saúde passou a ser um dos temas mais amplamente investigados em estudos com populações jovens.^1^^,^ 2 O interesse por investigações com foco nessa temática pode ser explicado, ao menos em parte, pela constatação de que tais comportamentos podem ser estabelecidos e incorporados ao estilo de vida em idades precoces^3^^,^ 4 e por sua ligação com fatores biológicos de risco^5^ e com a presença de doenças metabólicas ou cardiovasculares (DCV) já estabelecidas.^6^

A prevalência de simultaneidade de comportamentos de risco à saúde em adolescentes foi descrita em diversos estudos.^7^^-^ 17 Entretanto, constatou-se que os estudos desenvolvidos no Brasil, com exceção do levantamento feito por Farias Júnior et al.,^15^contaram com amostras muito específicas: alunos de colégio de aplicação^17^ e estudantes do turno diurno da rede pública de um município no Sul do Brasil.^16^ Portanto os resultados desses estudos não podem ser extrapolados para outras regiões do país devido aos contrastes socioeconômicos e culturais que sabidamente diferenciam a exposição a comportamentos de risco à saúde, conforme relatado por Nahas et al.^18^

Levantamentos epidemiológicos sobre simultaneidade de comportamentos de risco à saúde em adolescentes e seus fatores associados podem contribuir para identificar grupos de risco e para monitorar os níveis de saúde dessa população, o que pode subsidiar a elaboração de políticas públicas de promoção da saúde e tornar mais precoce as ações de intervenção para prevenção desses hábitos. Sendo assim, o objetivo do presente estudo foi analisar a prevalência e os fatores associados à simultaneidade de comportamentos de risco à saúde em adolescentes.

## Método

Trata-se de uma análise secundária dos dados de um levantamento epidemiológico transversal, de base escolar e abrangência estadual (Pernambuco, Brasil), denominado "Estilos de vida e comportamentos de risco à saúde em adolescentes: do estudo de prevalência à intervenção" O protocolo de investigação foi aprovado pelo Comitê de Ética em Pesquisa com Seres Humanos do Hospital Agamenon Magalhães, em atendimento às normas estabelecidas nas Resoluções 196 e 251 do Conselho Nacional de Saúde.

A população alvo, estimada em 352.829 sujeitos, segundo dados da Secretaria da Educação e Cultura do Estado de Pernambuco, foi constituída por estudantes do ensino médio matriculados na rede pública estadual, entre 14 e 19 anos. Para o cálculo do tamanho amostral foram empregados os seguintes parâmetros: intervalo de confiança de 95%; erro amostral de três pontos percentuais; prevalência estimada em 50% (opção adotada em função dos múltiplos fatores analisados no estudo); e o efeito de delineamento amostral, estabelecido em quatro vezes o tamanho mínimo da amostra. A partir desses parâmetros o tamanho amostral calculado foi de 4.217 estudantes.

Considerando o processo de seleção amostral, procurou-se garantir que os estudantes selecionados representassem a população alvo quanto à região geográfica (Metropolitana, Zona da Mata, Agreste, Sertão e Sertão do São Francisco), tamanho da escola e turno (diurno/noturno). A distribuição regional foi analisada a partir do número de estudantes matriculados em cada uma das 17 Geres (Gerências Regionais de Ensino). As escolas foram classificadas de acordo com o número de alunos matriculados no ensino médio e observaram-se os seguintes critérios: pequeno - menos de 200 alunos; médio - 200 a 499 alunos; e grande porte - 500 alunos ou mais. Os alunos matriculados nos períodos da manhã e da tarde foram agrupados numa única categoria (estudantes do período diurno). Todos os estudantes das turmas sorteadas foram convidados a participar.

Usou-se amostragem por conglomerado em dois estágios e adotou-se escola e turma como unidade amostral primária e secundária, respectivamente. No primeiro estágio, procedeu-se ao sorteio das escolas, de modo a incluir no mínimo uma escola de cada porte por Gere. No segundo estágio, foram sorteadas 203 turmas entre aquelas existentes nas escolas sorteadas no primeiro estágio.

Para coleta dos dados foi usada uma versão adaptada do questionário Global School-Based Student Health Survey (GSHS). Esse instrumento teve sua validade de face e conteúdo avaliada por especialistas (pesquisadores com experiência em estudos epidemiológicos com foco em condutas de saúde) e teve seus indicadores de validade concorrente e reprodutibilidade testados em estudo-piloto. Os indicadores de consistência de medida teste-reteste variaram de moderados a altos (coeficiente kappa=0,52 a 1,00)^19^^-^ 21 para a maioria dos itens. Os coeficientes de reprodutibilidade teste-reteste (coeficiente kappa) das medidas usadas no presente estudo foram: 0,86 para atividade física; 0,66 para o consumo de frutas; 0,77 para o consumo de hortaliças; 0,76 para o consumo de álcool; 0,62 para o uso de tabaco e 0,74 para o comportamento sedentário.

A coleta dos dados foi feita de abril a outubro de 2006. A aplicação dos questionários foi efetuada em sala de aula. Os estudantes foram assessorados por dois aplicadores previamente treinados, que esclareceram e auxiliaram no preenchimento dos dados. Todos os alunos foram informados que sua participação era voluntária e que os questionários não continham qualquer tipo de identificação pessoal. Os alunos também foram informados de que poderiam desistir em qualquer etapa da coleta de dados. Um termo de consentimento passivo foi usado para obter dos pais de estudantes com idade inferior a 18 anos permissão para que participassem do estudo. Estudantes com 18 anos ou mais assinavam o próprio termo e indicavam a sua concordância em participar do estudo.

A variável dependente (simultaneidade de comportamentos de risco à saúde) foi obtida a partir do somatório de cinco comportamentos de risco: baixo nível de atividade física (<300 minutos/semana); comportamento sedentário (>4 horas/dia); consumo ocasional de frutas e hortaliças (<1 vez por dia); consumo de bebidas alcoólicas (ter consumido bebidas alcoólicas nos últimos 30 dias) e tabagismo (ter fumado nos últimos 30 dias). A escolha desses fatores justifica-se por serem fatores modificáveis do estilo de vida que parecem estar mais fortemente associados a doenças crônicas não transmissíveis e que representam a maior carga global de adoecimento e mortalidade no mundo.^22^ Já o comportamento sedentário foi incluído por ser tratado como uma conduta distinta dos baixos níveis de atividade física e que apresenta uma alta prevalência na população, além de repercussões importantes na saúde do adolescente.^23^ Informações sobre descrição dessas variáveis poderão ser encontradas em publicações prévias.^19^^-^ 21 As respostas obtidas resultaram em um desfecho com zero (nenhum fator de risco presente) a cinco comportamentos de risco presentes. Posteriormente, para efeito de análise, a ocorrência de comportamentos de risco foi apresentada em quatro categorias (0, 1, 2, ≥3). As variáveis independentes foram: sexo; faixa etária (14-16 ou 17-19 anos); turno das aulas (diurnoou noturno); porte da escola (pequeno, médio ou grande); escolaridade materna (baixa: ≤8; média: 9-11; ou alta: ≥12 anos); situação ocupacional (trabalha/não trabalha); cor da pele (branca/não branca); região geográfica (Metropolitana, Zona da Mata ou Semiárido) e local de residência (urbana e rural).

O procedimento de tabulação dos dados foi efetuado em um banco de dados do programa EpiData Entry (versão 3.1). Para as análises, usou-se o programa Stata (versão 10). Na análise bivariada, recorreu-se à aplicação do teste de qui-quadrado para heterogeneidade e para tendência para verificar a prevalência de simultaneidade de comportamentos de risco à saúde por categorias das variáveis independentes.

Para analisar possíveis associações entre variável independente e dependente foi feita análise de regressão logística ordinal, com modelo de *odds* proporcionais. A suposição de proporcionalidade foi verificada pelo teste de razão de verossimilhança e a significância dos coeficientes por meio do teste de Wald. As análises foram feitas em duas etapas: primeiro, regressões simples das variáveis independentes em relação ao desfecho. Em seguida, análise multivariável com vistas a determinar se os fatores demográficos e relacionados à escola estavam ou não associados ao desfecho. Todas as variáveis independentes entraram no mesmo nível de análise no modelo multivariável e foram excluídas por método *backward stepwise*. Adotou-se um valor *p* inferior a 0,2 como critério para exclusão das variáveis durante as etapas da modelagem. Esses resultados são apresentados na forma de razões de *odds* e respectivos intervalos de confiança.

Após a seleção das variáveis que iriam constituir o modelo de regressão, testou-se a existência de possível colinearidade entre as covariáveis região geográfica (Metropolitana, Zona da Mata ou Semiárido) e local de residência (urbana e rural). Não foi identificada relação linear (valores VIF <10) entre essas duas variáveis.

## Resultados

Dos adolescentes presentes nas turmas sorteadas nas 76 escolas investigadas (4.269), 55 se recusaram a participar e sete foram excluídos devido a dados incompletos ou inconsistentes no questionário. A amostra final ficou constituída por 4.207 adolescentes (59,8% moças), entre 14 e 19 anos (média de 16,8 anos; DP=1,4). Outras características da amostra estão apresentadas na Tabela 1. Dentre as variáveis analisadas no estudo, com exceção da escolaridade materna (6,1%), as taxas de questões sem resposta não ultrapassou 2,0%.

**Tabela 1:** Características da amostra por sexo

Variável	Todos			Rapazes			Moças	
	%	n		%	n		%	n
*Faixa etária*									
14‐16 anos	42,0	1.766		35,4	598		46,4	1.165
17‐19 anos	58,0	2.441		64,6	1.089		53,6	1.346

*Turno*									
Diurno (manhã/tarde)	57,6	2.414		53,9	908		60,0	1.506
Noturno	42,4	1.780		46,1	778		40,0	1.002

*Escolaridade materna*									
≤ 8	72,5	2.865		69,4	1.086		74,5	1.771
9‐11	21,1	833		22,5	352		20,2	480
≥ 12	6,4	253		8,1	127		5,30	126

*Cor da pele*									
Branca	25,2	1.057		24,8	417		25,5	639
Não branca	74,8	3.136		75,2	1.262		74,5	1.866

*Região geográfica*									
Metropolitana	41,8	1.757		39,8	670		43,2	1.084
Zona da Mata	17,7	743		18,1	306		17,3	434
Semiárido	40,6	1.707		42,1	711		39,5	993

Local de residência									
Urbana	79,0	3.294		78,1	1.311		79,5	1.983
Rural	21,0	877		21,9	367		20,5	510

*Porte da escola*									
Pequeno	8,9	376		9,0	152		8,80	221
Médio	25,8	1.084		27,0	456		25,0	628
Grande	65,3	2.747		64,0	1.079		66,2	1.662

Situação ocupacional									
Não trabalha	78,5	3.276		69,2	1.157		84,8	2.119
Trabalha	21,5	895		30,8	514		15,2	381
Baixo nível de atividade física	65,1	2.731		57,5	971		70,2	1.754
Exposição ao comportamento sedentário	18,7	782		16,5	277		20,2	504
Consumo ocasional de frutas e/ou hortaliças	51,4	2.145		53,5	893		50,0	1.248
Consumo de bebidas alcoólicas	30,4	1.273		38,6	648		24,8	622
Tabagismo	7,6	320		9,8	165		6,2	155

Na Figura 1 estão apresentadas as prevalências de exposição aos cinco comportamentos de risco à saúde focalizados no presente estudo. Os resultados referentes a esses comportamentos não serão explorados neste estudo, pois já foram apresentados, de forma isolada, em investigações prévias.^19^^-^ 21 A Figura 2 apresenta a prevalência de simultaneidade de exposição a comportamentos de risco à saúde observada na amostra por sexo.

**Figura 1:** Prevalência de comportamentos de risco à saúde em adolescentes escolares do ensino médio do Estado de Pernambuco, Brasil, 2006.

**Figura 2:** Prevalência de simultaneidade de comportamentos de risco à saúde em adolescentes escolares do ensino médio do Estado de Pernambuco, Brasil, 2006.

Na análise bivariada, identificou-se que a proporção de adolescentes expostos a três ou mais comportamentos de risco de forma simultânea foi estatisticamente maior entre os estudantes mais velhos (17-19 anos), adolescentes com maior escolaridade materna, estudantes que residem em área urbana e aqueles que moram na Região Semiárido em comparação com seus pares (Tabela 2).

**Tabela 2:** Prevalência de simultaneidade de comportamentos de risco à saúde de acordo com variáveis demográficas, socioeconômicas, relacionadas à escola e a divisões regionais em adolescentes do ensino médio de Pernambuco, 2006

Variáveis	Simultaneidade de comportamentos de risco (CR)											
	0 CR			1 CR			2 CR			≥3 CR		*p*-valor
	%	n		%	n		%	n		%	n	
**Fatores demográficos**												
*Sexo*												
Masculino	10,9	180		29,9	491		34,8	571		24,4	400	<0,083
Feminino	9,7	240		32,1	794		39,2	968		19,0	470	

*Idade*												
14‐16 anos	10,5	183		32,2	560		38,3	666		19,0	331	0,028
17‐19 anos	9,9	237		30,6	730		36,8	876		22,7	540	

*Escolaridade materna*												
≤ 8 anos	9,9	277		32,3	907		38,8	1.091		19,0	535	0,008
9‐11 anos	11,2	92		28,6	234		33,5	274		26,7	219	
≥ 12 anos	11,2	28		26,5	66		35,7	89		26,5	66	

*Cor da pele*												
Branco	8,8	91		33,8	350		35,4	366		22,0	227	0,732
Não branco	10,6	326		30,4	934		38,1	1.172		20,9	644	

*Região geográfica*												
Metropolitana	12,0	207		32,0	551		35,3	609		20,7	356	<0,001
Zona da Mata	9,5	69		33,7	246		40,6	296		16,2	118	
Semiárido	8,6	144		29,5	493		38,1	637		23,8	397	

*Local de residência*												
Urbana	10,1	328		30,8	994		36,6	1.181		22,5	728	0,009
Rural	9,9	86		33,7	292		40,4	350		15,9	138	

**Fatores relacionados à escola**												
*Turno*												
Diurno	10,6	252		31,5	749		36,6	870		21,3	507	0,522
Noturno	9,7	168		31,1	541		38,4	669		20,8	363	

*Porte da escola*												
Pequeno	10,6	39		27,7	102		39,4	145		22,3	82	0,085
Médio	11,0	117		35,6	378		35,2	374		18,2	193	
Grande	9,8	264		30,1	810		38,0	1.023		22,1	596	
**Fatores socioeconômicos**												
*Situação ocupacional*												
Não trabalha	9,6	308		31,5	1.017		37,8	1.219		21,1	682	0,209
Trabalha	12,5	109		30,2	263		36,0	313		21,3	186	

Na Tabela 3 estão apresentados os resultados da análise da regressão logística ordinal para a simultaneidade de comportamentos de risco à saúde de acordo com os fatores demográficos e relacionados à escola. Na análise ajustada, constatou-se que idade, situação ocupacional, escolaridade materna, região geográfica e local de residência foram estatisticamente associados a maior simultaneidade de comportamentos de risco à saúde.

**Tabela 3:** Regressão logística ordinal para simultaneidade de comportamentos de risco à saúde e variáveis demográficas, socioeconômicas, relacionadas à escola e por divisão regional em adolescentes do ensino médio de Pernambuco, 2006

Variáveis	Simultaneidade de comportamentos de risco					
	OR bruta	IC95%	*p*	OR ajustada	IC95%	*p*
*Sexo*						
Masculino	1			1		
Feminino	0,90	0,80‐1,00	0,060	0,91	0,81‐1,03	0,140

*Idade*						
14‐16 anos	1			1		
17‐19 anos	1,13	1,01‐1,27	0,028	1,17	1,04‐1,32	0,008
*Escolaridade materna*						
≤8 anos	1		0,004	1		0,009
9‐11 anos	1,21	1,05‐1,40	0,010	1,21	1,04‐1,40	0,011
≥12 anos	1,26	0,99‐1,60	0,058	1,21	0,95‐1,55	0,121

*Cor da pele*						
Branco	1			Excluído		
Não branco	0,99	0,87‐1,13	0,927			

*Região geográfica*						
Metropolitana	1			1		
Zona da Mata	0,97	0,83‐1,14	0,731	1,07	0,90‐1,26	0,435
Semiárido	1,27	1,13‐1,44	<0,001	1,39	1,22‐1,59	<0,001

*Local de residência*						
Urbana	1			1		
Rural	0,83	0,73‐0,95	0,008	0,78	0,68‐0,91	0,001

*Turno*						
Diurno	1			Excluído		
Noturno	1,04	0,93‐1,16	0,542			
*Porte da escola*						
Pequeno	1		0,084	Excluído		
Médio	0,76	0,61‐0,94	0,013			
Grande	0,97	0,79‐1,19	0,782			
*Situação ocupacional*						
Não trabalha	1			1		
Trabalha	0,93	0,81‐1,06	0,285	0,86	0,74‐0,99	0,040

Verificou-se que os adolescentes mais velhos (17-19 anos) apresentaram chance 17% superior de exposição simultânea a mais de três comportamentos de risco à saúde quando comparados com os mais jovens. Os estudantes que relataram trabalhar tinham chance 14% inferior de apresentar mais de três comportamentos de risco quando comparados com aqueles que não trabalhavam. Por outro lado, os adolescentes que referiram mães com escolaridade intermediária (9-11 anos) tinham chance 21% superior de apresentar maior simultaneidade de comportamentos de risco quando comparados com aqueles que relataram menor escolaridade materna (≤8 anos).

A chance de coocorrência de maior número de comportamentos de risco à saúde foi 22% inferior entre os adolescentes que relataram residir na área rural em comparação com aqueles que residiam em área urbana. Adolescentes que referiram morar na Região Semiárido apresentaram chance 39% superior de exposição a múltiplos comportamentos de risco à saúde em comparação com os adolescentes residentes na região metropolitana.

## Discussão

Os resultados do presente estudo evidenciam que a prevalência de exposição simultânea a comportamentos de risco à saúde entre os adolescentes pernambucanos foi elevada, assim como observado em estudos congêneres.^8^^,^ 10 ^-^ 12 Outro resultado importante foi a identificação de cinco fatores significativamente associados à maior coocorrência desses comportamentos, a saber: faixa etária, escolaridade materna, região geográfica, situação ocupacional e local de residência.

Os resultados deste levantamento indicaram que 58,5% dos adolescentes estavam expostos simultaneamente a dois ou mais comportamentos de risco, como observado em estudo conduzido no município de João Pessoa, PB.^15^ A importância desse achado recai sob o fato de que problemas de saúde podem ser causados por conjunto de comportamentos de riscos agregados, a exemplo do câncer de garganta, que pode ser explicado pela adoção simultânea de dois hábitos (tabagismo e alcoolismo), como destaca a OMS.^24^

No presente estudo, a exposição simultânea a maior número de comportamentos de risco à saúde foi mais elevada entre os adolescentes mais velhos. Como pode ser visto em estudos disponíveis na literatura, a prevalências de exposição simultânea a comportamentos de risco à saúde aumenta com a idade.^8^^,^ 13 ^,^ 25 O que pode ser explicado pela aquisição de maior autonomia, independência social e econômica do adolescente,^26^ o que favorece o acesso a locais que vendem bebidas alcoólicas, cigarros e outras drogas.

Chama atenção no presente estudo a associação entre a escolaridade materna intermediária (9-11 anos) e a maior ocorrência de comportamentos de risco à saúde entre os adolescentes. Isso é curioso porque quanto maior o nível de escolaridade, supostamente maior conhecimento a mãe teria sobre os benefícios de se adotarem estilos de vida mais saudáveis e, consequentemente, uma maior possibilidade de fomentar mais apoio a seus filhos.^27^ Uma das possíveis explicações reside no fato de que níveis de escolaridade mais elevados são daquelas mães que provavelmente trabalham fora de seus domicílios e, por conseguinte, despendem menor tempo com os filhos adolescentes.

Verificou-se também que os adolescentes que referiram exercer atividades laborais tinham chances mais baixas de exposição simultânea a maior número de comportamentos de risco à saúde quando comparados com os que não trabalhavam. Numa sociedade em que os jovens enfrentam grandes desafios para inserção no mercado de trabalho, é possível supor que os jovens que exercem alguma atividade laboral tenham maior autoestima, autonomia e responsabilidade pessoal, características que podem favorecer a adoção de condutas mais saudáveis.

Adolescentes que residem na Região Semiárido apresentam um aumento de 39% na chance de exposição simultânea a maior número de comportamentos de risco à saúde quando comparados aos seus pares que moram na região metropolitana. Estudos comparativos com análise de exposição simultânea a hábitos de vida são escassos, o que impossibilita comparações. Entretanto, Matsudo et al.^28^ fizeram um estudo no Estado de São Paulo e observaram que as pessoas do litoral eram mais ativas do que aquelas que moravam no interior. Isso pode estar relacionado à baixa oferta de equipamentos de lazer e instalações para atividades físicas em cidades interioranas. Assim como pode estar relacionado à disponibilidade, acessibilidade e qualidade de conservação dos alimentos nessa região, onde há reconhecida escassez de recursos hídricos fundamentais tanto para produção como para processamento de alimentos frescos.

Por outro lado, adolescentes que moram na área rural tinham uma diminuição de 22% na chance de exposição simultânea a maior número de comportamentos de risco à saúde quando comparados com os que residiam em área urbana. Isso pode ser explicado pelas características específicas dos tipos de atividades nas áreas rurais, as quais demandam maior gasto energético para o cumprimento dessas tarefas (ex. agricultura extensiva e familiar, pecuária, extrativismo vegetal, extrativismo mineral, entre outros),^29^além do maior acesso a alimentos como cereais e derivados (feijão, arroz e milho), raízes (batata, mandioca e outros), os quais são essencialmente produtos do cultivo da agricultura familiar, além do menor acesso a refeições prontas e misturas industrializadas.^30^

A carência de estudos semelhantes dificulta a comparação dos achados do presente estudo. O que foi encontrado na literatura ficou limitado às investigações que avaliaram a associação desses fatores com a exposição isolada a um ou outro comportamento de risco. Os estudos congêneres disponíveis^13^^-^ 17adotaram procedimentos metodológicos bastante diversificados, particularmente quanto a tipo, quantidade e definição das variáveis caracterizadoras de risco.

A generalização dos resultados do presente estudo deve ser efetuada com cautela, pois somente adolescentes estudantes da rede pública estadual foram incluídos. É possível assumir que os resultados sejam diferentes em amostras de adolescentes estudantes de escolas privadas e entre aqueles que não estão engajados no sistema formal de educação. Por outro lado, a decisão de não incluir escolas do sistema privado no planejamento amostral foi decorrente da constatação de que mais de 80% dos adolescentes pernambucanos estavam matriculados em escolas públicas estaduais.

Vale ressaltar que as prevalências apresentadas neste estudo revelam um cenário observado já há algum tempo e, portanto, a interpretação desses parâmetros deve ser efetuada com atenção, pois algumas mudanças sociais e demográficas que ocorreram na região Nordeste durante esse período podem ter afetado esses indicadores. Por outro lado, não é plausível admitir que as associações que foram identificadas e relatadas neste estudo seriam diferentes em decorrência da eventual modificação na prevalência de algum fator.

Apesar dos bons níveis de reprodutibilidade do instrumento, não se pode descartar a possibilidade de viés de informação, já que adolescentes tendem a superestimar ou, e em outras ocasiões, subestimar a exposição a condutas de risco.

No entanto, os achados deste levantamento adicionam importantes evidências ao corpo de conhecimento disponível sobre prevalência e os fatores associados à simultaneidade de comportamentos de risco à saúde em adolescentes. Além disso, o estudo foi conduzido com uma amostra relativamente grande, representativa dos estudantes do ensino médio da rede pública no Estado de Pernambuco. Acredita-se que as evidências apresentadas neste estudo podem auxiliar na identificação de subgrupos mais vulneráveis e contribuir consequentemente para tomada de decisões e para o planejamento de estratégias de intervenção adequadas. Além disso, podem suscitar o desenvolvimento de outras investigações.

Diante dos achados apresentados, pode-se concluir que há uma grande parcela de adolescentes expostos a comportamentos de risco à saúde de forma simultânea. Constatou-se também que os adolescentes com maior faixa etária, com mães de níveis de escolaridade intermediários e residentes na Região Semiárido apresentavam chance mais elevada de exposição simultânea a maior número de comportamentos de risco à saúde, o que configura os subgrupos de maior risco. Ao passo que os adolescentes trabalhadores e aqueles que residiam em áreas rurais apresentaram menor chance de exposição simultânea a maior número de comportamentos de risco.
